# Assessment of closely related *Mycobacterium tuberculosis* variants with different transmission success and in vitro infection dynamics

**DOI:** 10.1038/s41598-021-90568-x

**Published:** 2021-05-26

**Authors:** Estefanía Abascal, Charlotte Genestet, Ana Valera, Marta Herranz, Miguel Martinez-Lirola, Patricia Muñoz, Oana Dumitrescu, Darío García de Viedma

**Affiliations:** 1 Servicio de Microbiología Clínica y Enfermedades Infecciosas, Hospital General Universitario Gregorio Marañón, C/Dr. Esquerdo 46, 28007 Madrid, Spain; 2 Instituto de Investigación Sanitaria Gregorio Marañón (IiSGM), Madrid, Spain; 3 CIRI - Centre International de Recherche en Infectiologie, Ecole Normale Supérieure de Lyon, Inserm U1111, CNRS UMR5308, Université Claude Bernard Lyon-1, 69007 Lyon, France; 4 Laboratoire de bactériologie, Institut des Agents Infectieux, Hospices Civils de Lyon, 69317 Lyon Cedex 04, France; 5 CIBER Enfermedades Respiratorias (CIBERES), Madrid, Spain; 6 Complejo Hospitalario Torrecárdenas, Almería, Spain; 7 Departamento de Medicina, Universidad Complutense de Madrid, Madrid, Spain

**Keywords:** Microbiology, Molecular biology, Diseases

## Abstract

Whole genome sequencing (WGS) is able to differentiate closely related *Mycobacterium tuberculosis* variants within the same transmission cluster. Our aim was to evaluate if this higher discriminatory power may help identify and characterize more actively transmitted variants and understand the factors behind their success. We selected a robust MIRU-VNTR-defined cluster from Almería, Spain (22 cases throughout 2003–2019). WGS allowed discriminating, within the same epidemiological setting, between a successfully transmitted variant and seven closely related variants that did not lead to secondary cases, or were involved in self-limiting transmission (one single secondary case). Intramacrophagic growth of representative variants was evaluated in an in vitro infection model using U937 cells. Intramacrophage multiplication ratios (CFUs at Day 4/CFUs at Day 0) were higher for the actively transmitted variant (range 5.3–10.7) than for the unsuccessfully transmitted closely related variants (1.5–3.95). Two SNPs, mapping at the DNA binding domain of DnaA and at *kdpD*, were found to be specific of the successful variant.

## Introduction

It is estimated that around a quarter of the world’s population is infected by *Mycobacterium tuberculosis* (MTB). Nevertheless, only 5–10% of the cases progress to active tuberculosis (TB) and are responsible for the transmission to secondary cases. TB can show a wide range of symptoms and clinical severity and the dynamics of its transmission from an infectious case may differ extensively^1^.

Historically, it has been assumed that, due to the low genetic diversity within members of the *M. tuberculosis* complex (MTBC), differences in infection outcomes or dynamics of transmission are linked to the host or environmental factors. However, varying degrees of diversity have been broadly demonstrated for MTBC members, which could also have a potential role in the differences described at clinical or epidemiological levels^2^.

To characterize the diversity of MTBC we may tune our analysis, depending on the discriminatory power of the genotyping tools we apply. Lower discriminatory power techniques allow classifying MTB in eight lineages^3–5^ that differ not only in their genetic background but also in their geographical distribution^6^, transmission efficiency^7,8^, and invasiveness, virulence, etc^9^.

Over the last 25 years, molecular epidemiology approaches have allowed to extend our knowledge on TB transmission and helped improve control measures^10^. The use of infection models allows determining correlations between certain strains that successfully transmit and their ability to replicate within macrophages^11–13^.

Whole genome sequencing (WGS) has improved the precision of assessing diversity between MTB isolates, revealing that the standard genotyping tools used until now, including those with greater discriminatory power such as the 24-loci MIRU-VNTR, cannot define clusters accurately^10^.

In a population-based MIRU-VNTR systematic analysis including all the TB cases during fourteen consecutive years in the province of Almería (SouthEast Spain), with the majority of TB cases involving migrants, we observed how, within a theoretically robust MIRU-VNTR defined cluster, Cluster 113 (lineage 4.3.2, pansusceptible)^14^, it was possible to discriminate by WGS analysis between variants that were successfully transmitted and other closely related variants not responsible of secondary cases or involved in self-limiting small transmission events^14–16^.

Cluster 113 is a good example of how WGS allows to accurately reinterpret MIRU-VNTR defined clusters in epidemiologically complex populations with a high rate of migrants^17,18^. The most likely interpretation of the topology of the WGS-based network, supported on a strain-specific-PCR-based surveillance in Morocco and the available epidemiological data, is that these variants probably emerged in Morocco after the strain had been long circulating in that country, and were independently exported from Morocco to Almería, Spain^14^. Once exported, certain variant was successfully transmitted in Almería, whereas others did not cause secondary cases.

The purpose of this study is to extend the previous efforts based on lower resolution genotyping approaches to the more refined WGS data, to find relationships between diversity and infectivity/transmission efficiency. Based on the subtle differences revealed by WGS between closely related variants within the MIRU-VNTR defined Cluster 113, we will assess whether bacterial factors may have a role in the differential transmission efficiency by coupling WGS analysis with the evaluation of these variants in a macrophage infection model.

## Materials and methods

### Macrophage infection model

We assessed the infectivity and multiplication ability of the selected variants from Cluster 113, represented by successfully transmitted strains or not transmitted (not causing secondary cases) variants, using an in vitro macrophage infection model^19^.

**Figure 1:**
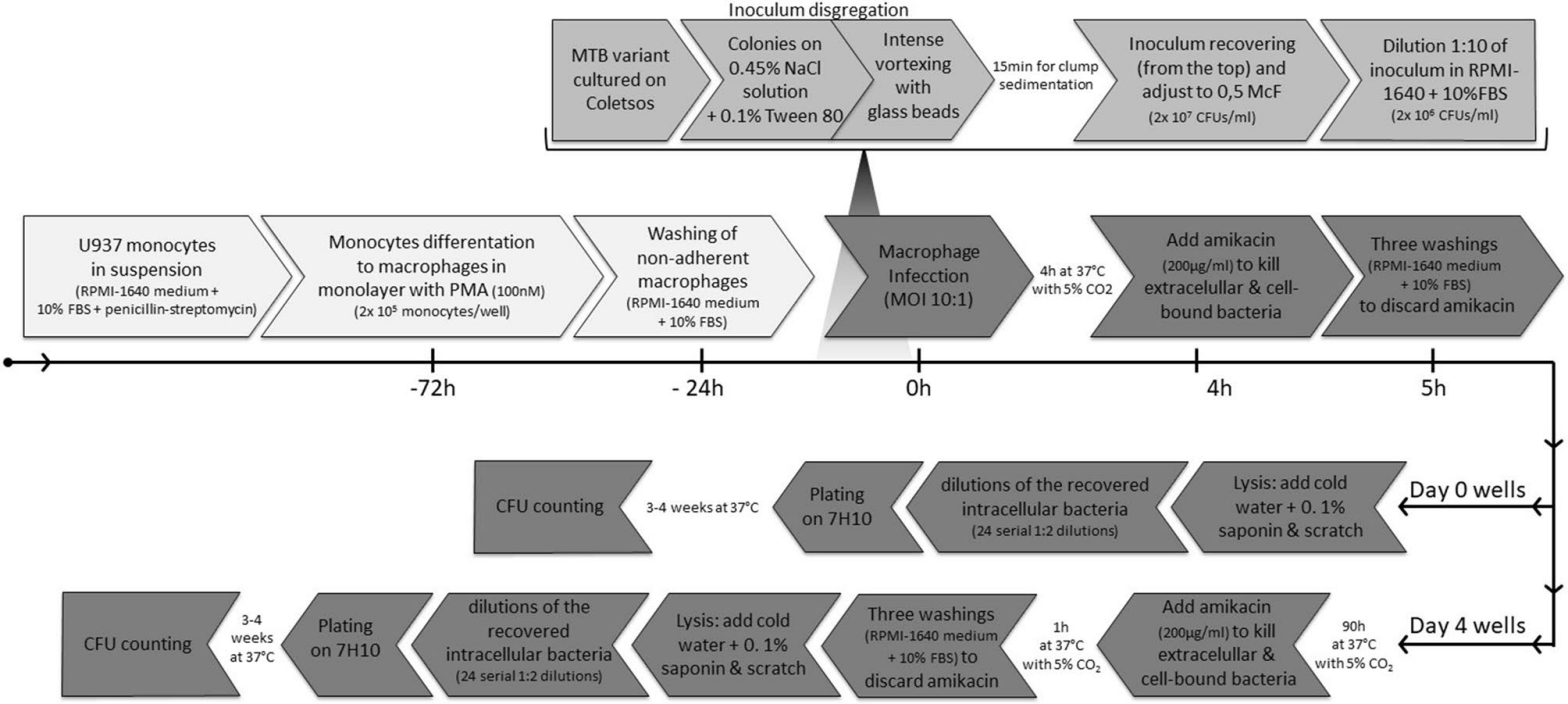
Flow chart indicating the steps in the infection model.

Briefly (Fig. 1), U937 monocytic cells (monocytic cell line from pleural effusion (ATCC® CRL-1593.2™)) were grown in suspension in RPMI-1640 medium supplemented with 10% fetal bovine serum (FBS) plus penicillin and streptomycin at final concentrations of 100 U/mL and 0.1 mg/ml, respectively (1% penicillin–streptomycin solution, Sigma-Aldrich, St. Louis, Missouri, USA) to minimize contamination during cell subculture. Monocytes were differentiated to macrophages by adding phorbol 12-myristate 13-acetate at a final concentration of 100 nM and left for 48 h, following the standard protocol of differentiation of macrophages by PMA stimulation^20^. Following differentiation, the macrophages attached to the bottom of the well in a monolayer were washed once with RPMI media + 10% foetal bovine serum to remove non-adherent macrophages. After 24 h, cells were infected, in antibiotic-free medium, with the MTB variants (one single variant tested per well) at a 10:1 (bacteria to cells) multiplicity of infection. For inoculum preparation, MTB variants were grown on egg-based Coletsos medium, collected in exponential phase, at the beginning of colony formation on Coletsos medium, suspended in 3 mL of sterile 0.45% NaCl solution + 0.1% Tween 80 and homogenized by exhaustive vortexing with glass beads in 15 mL glass tubes. The preparation was left for 15 min to allow clumps to sediment. Next, homogenized bacteria suspensions were recovered from the upper part and the concentration was adjusted bacterial suspension at 0.5McF, corresponding to approximately 2 × 10^7^ CFU/mL according to our lab optimization tests. After four hours of infection, the infected cells were incubated for one hour with amikacin (final concentration of 200 µg/mL) to kill extracellular and cell-membrane bound bacteria, followed by three washings to discard amikacin before macrophage lysis and bacteria plating.

To evaluate invasiveness (Day 0), cells were lysed, after removing amikacin, by adding cold water + 0.1% saponin and scratched thoroughly. Intracellular mycobacteria were recovered and 24-fold 1:2 serial dilutions performed. Next, 7 µL droplets of each dilution were plated on 7H10 agar, left for 3–4 weeks at 37 °C and then visually inspected to determine colony-forming units (CFUs).

To measure intramacrophage multiplication ratios (CFUs at Day 4/CFUs at Day 0) amikacin was added at Day 4 post-infection to remove extracellular bacteria and recover intracellular bacteria. Plating and CFU count analysis were performed as described above.

All variants were included in every infection round, to rule out biases due to methodological inter-experiment variability. Additionally, a duplicate infection of every isolate was performed in two different wells at each endpoint to discard intra-experiment deviations (mean results were considered for the statistical analysis). Infections were repeated three times for validation purposes.

### Statistical analysis

Statistical analyses were performed using GraphPad Prism 5. Bacterial counts (Day 0 and Day 4/Day 0 ratio) were expressed as median values ± interquartile range and compared using the Kruskal–Wallis test followed by Dunn's post-hoc test. A *p* value < 0.05 was considered statistically significant.

### Analysis of variant specific single-nucleotide polymorphisms

SNPs were identified from WGS data in a previous analysis (sequences were deposited in www.ebi.ac.uk, study ID: PRJEB23664)^14^. The new cluster-113-isolates included in this study were processed and sequenced following the same procedures, including the same SNP calling pipelines (sequences were deposited in www.ebi.ac.uk (PRJEB23664, PRJEB25814; ERS6279864 (SAMEA8595229) for the sequences from previous studies^14^ and ERS6279865 (SAMEA8595230) and ERS6279866 (SAMEA8595231) for the new sequences). We considered heterozygous calls when two alternative alleles (the one corresponding to the reference and the alternative allele found in our strain) were simultaneously called in a position.

For all specific SNPs in successfully transmitted variants (present in it but absent in closely related variants), which were coding (calls mapping in genes) and non-synonymous, a review from the literature was performed to determine their potential involvement in MTB virulence/infectivity.

## Results and discussion

In a previous study^14^, 24-locus-MIRU-VNTR defined clusters from a population-based study carried out in Almería (Spain) (including all the culture-positive TB cases diagnosed in the entire province during 14 consecutive years; 2003–2017), were analysed by WGS, which in some clusters resulted in the division of closely related isolates (≤ 5 SNPs) and several variants with a higher diversity (> 10 SNPs).

In the present study, we examined the clusters with marked asymmetry as per the WGS analysis; one MTB variant was found in a large number of cases, while other/s more distantly related variant/s were identified in a smaller number of cases. We used the number of cases infected by each variant as proxy of differential transmission success.

**Figure 2:**
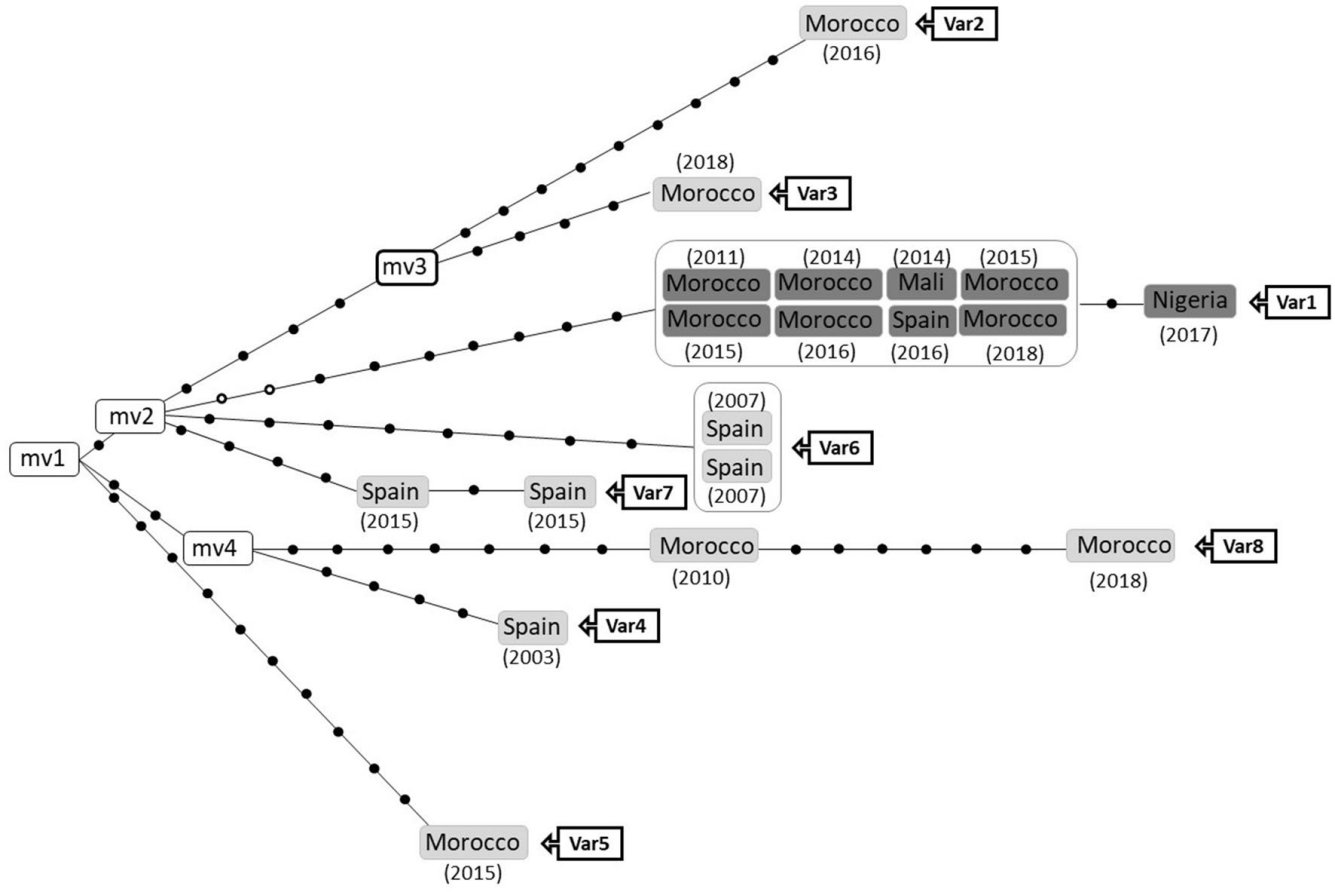
Each box represents a patient, differentiating the ones infected by the successfully transmitted variant (Variant 1, coloured in dark grey) from those infected by unsuccessfully transmitted closely related variants (in light grey). Patient´s origin is indicated within the box and the year of diagnosis in brackets. When two or more cases share identical sequences (0 SNPs between them) they are boxed, surrounded by a line. Each black dot represents a SNP. The white circles in Variant 1 branch correspond to non-fixed alleles, found in heterozygosis in the index case (Morocco-2011) but in homozygosis in the remaining cases. In the white boxes the name of the variant is indicated (Var). *mv* median vectors (corresponding to not-sampled nodes).

Cluster 113^14^ was selected as the source of MTB closely related variants with differential transmission efficacy. We first updated its composition, incorporating WGS data of the new cases identified after its initial description in 2017, in a population-based MIRU-VNTR systematic analysis including all the TB cases in the province during fourteen consecutive years^14^. The updated cluster network (Fig. 2) showed 19 cases, mainly Moroccan migrants (11 cases) and a limited representation of cases from Spain (six cases), Mali (one case), and Nigeria (one case). The network distributed the cases in seven branches, despite sharing identical MIRU-VNTR patterns. The branch with most cases involved Variant 1 (9 cases with 0–1 SNPs pairwise distances, most likely due to uncontrolled recent transmission). Four branches (involving Variants 2–5) were dead-ends, i.e., single cases not causing secondary cases. The remaining three branches (variants 6–8) represented, self-limiting, short transmission events with only two cases.

We tested the behaviour of two representatives for the actively transmitted Variant 1 (Morocco-2011, index case, and Morocco-2014) in an in vitro macrophage infection model (Fig. 1). Variants 2, 5, and 8-2010 (differing 19–22 SNPs from the successful variant) were selected as non-transmissible controls, because these variants corresponded to cases diagnosed in 2010, 2015 and 2016, respectively. Therefore, despite having had enough time since their first detection until today to led to transmission events, they did not cause any secondary case (or only one, for Variant 8) (Fig. 2).

**Figure 3:**
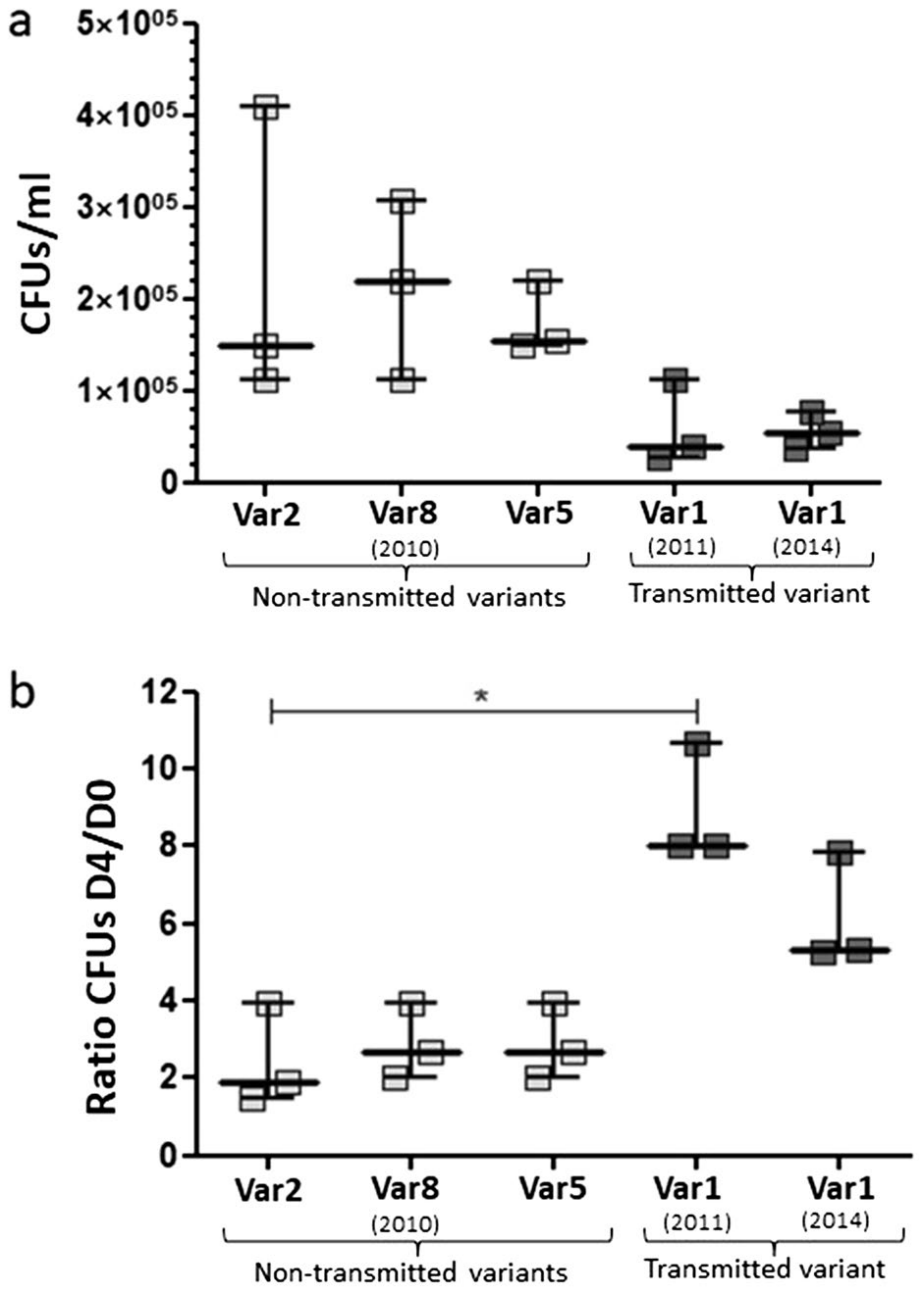
(**a**) Macrophage invasiveness (CFUs at Day 0) for the five selected variants. Each square represents the mean value of an experiment. Lines and error bars indicate median values ± range. (**b**) Intramacrophage multiplication ratios (CFUs at Day 4/CFUs at Day 0) for the five selected variants. Each square represents the mean value of an experiment. Lines and error bars indicate median values ± interquartile range. *p < 0.05.

We first assessed dissimilarities in invasiveness. No statistical differences were found between Variant 1 and the non-transmitted control variants (Fig. 3a). Certain outbreak strains, namely CDC1551, demonstrated high transmissibility but were not found to be more virulent^21,22^.

Next, we evaluated their intramacrophage multiplication rates. We observed a statistically significant superiority of Variant 1 based on intracellular growth (Fig. 3b). The multiplication ratios for Variant 1 were two–four folds higher than the obtained for the non-transmitted variants [ratios ranging from 5.3 to 10.7 vs. from 1.5 to 3.95, respectively; *p* = 0.0244 for Variant 1 (2011)]. The higher intramacrophage multiplication observed for Variant 1 led us to a further detailed analysis to determine if the differential SNPs found between this variant and the control ones could explain that behaviour. Among the nine specific SNPs identified exclusively in Variant 1 (Table 1), seven were homozygous calls for all isolates, but the remaining two were heterozygous calls in the index case (Morocco-2011) and were thus fixed in the rest of the cases. Two SNPs were located in intergenic regions, which could play a role, if they relate to regulatory elements. For those intragenic, we focused on the four corresponding to non-synonymous mutations. Two of them mapped on genes without a described function (the Rv0090 and Rv1517 hypothetical proteins), while the other two may have a functional meaning (Rv0001, alias *dnaA*, and Rv1028c, alias *kdpD*).

**Table 1:** Specific SNP annotation for the successfully transmitted Cluster 113 variant.

SNP position	Nucleotide change	Allele freq index^a^	Gene	Gene coordinates	Gene direction	Essential character	Protein length	Aminoacid change	Function
1163	C > T	0.29	Rv0001 (dnaA)	1…1524	+	Essential	507	T > I	Chromosomal replication initiation protein
1623635	G > A	0.5	Rv1444c	1623287…1623697	−	Non-essential	136	Synonymous	Hypothetical protein
99141	T > G	> 0.9	Rv0090	98480…99250	+	Non-essential	256	L > R	Hypothetical protein
1151418	C > T	> 0.9	Rv1028c (kdpD)	1149104…1151686	−	Non-essential	860	R > Q	Sensor protein KdpD
1163957	C > T	> 0.9	IG1057 (Rv1040c-Rv1041c)	1163377…1164571					
1709423	G > T	> 0.9	Rv1517	1708871…709635	+	Non-essential	254	G > C	Hypothetical protein
2142832	C > T	> 0.9	Rv1895	2142521…2143675	+	Non-essential	384	Synonymous	Possible dehydrogenase
3659433	G > A	> 0.9	Rv3276c (purK)	3658635…3659924	−	Non-essential	429	Synonymous	Phosphoribosylaminoimidazole carboxylase ATPase subunit
4314130	T > C	> 0.9	IG3905 (Rv3840-Rv3841)	4313981…4314177					

^a^Allele frequency in all secondary cases was > 0.9.

*dnaA* encodes a key protein for triggering the chromosomal replication machinery at *ori*C through interactions with the DnaA-boxes, which assembles a nucleoprotein complex responsible for the ATP-dependent opening of the double strand of DNA^23^. The differential SNP found between this variant and the control ones maps at residue 388 of the DNA binding domain of DnaA^24^ and causes a threonine (polar uncharged amino acid) to isoleucine (hydrophobic amino acid) substitution. Although modelling data are required to interprete the meaning of this substitution, we might speculate that the nature of this substitution may improve DnaA-oriC interactions. The fact that the SNP mapping in *dna*A was initially detected in heterozygosis in the index case and was then fixed in all secondary cases, may reinforce the advantageous character of this substitution.

*kdpD* is one of the two genes that constitute the *kdpDE* operon, one of the scarce two-component regulatory systems (2CRSs) found in MTB, and it encodes its sensor protein^25^. It has been shown that strains with deletions in *kdp*DE, and in some other MTB 2CRSs, increase their virulence in an immunodeficient mouse model^26^.

The KdpDE 2CRS system is involved in the detection and response of pH and K + changes (related with turgor pressure, regulation of cytoplasmic pH, osmolarity, transmembrane electrical potential, etc.) by modulating the expression of a K + transport system^27^. This system may have a role in MTB efficiency to survive acidic environments, e.g., phagosome or autophagosome vacuoles^27^. The specific SNP harboured by Variant 1 at the N-terminal domain of this protein implies an arginine (positively charged amino acid) to glutamine (polar uncharged amino acid) substitution. The domain interacts with two lipoproteins, LprF and LprJ, to form a ternary complex that modulates the sensing ability of KdpD^28^. Thus, the substitution described for Variant 1 may affect the binding affinity of Lpr and the subsequent expression of the K + transport system, and consequently the modulation of pH and K + uptake. However, these are mere hypothesis that require further validation/evidence.

Folkvardsen et al.^15^ identified a specific SNP in a 2CRS from a strain responsible for one of the major transmission events worldwide—currently active—not present in a closely related variant that caused few secondary cases. This SNP is located in the *tcrY*^15^ gene, which also codes for the sensor TcrXY protein of a 2CRS system. It has also been proven that deletions in this 2CRS system results in a hypervirulent phenotype in SCID mice^26^. We must admit that several of the observations related to the virulence of mutants in 2CRS systems were obtained from immunocompromised models and might not be transferable to an inmunocomponent system, such as the cases from which Variant 1 was obtained.

In summary, we have identified two specific SNPs in Variant 1, located in *dnaA* and *kdpD,* not found in other closely related non-transmitted variants. The changes caused by these SNPs may be associated to the higher infectivity shown by Variant 1 in macrophages. We cannot fully exclude the role of other potentially undetected mutations. This is because repetitive regions, including, among others, the PE and PPE genes, must still be systematically excluded from any Illumina-based WGS analysis. It causes the existence of black boxes with unknown diversity^29^, in regions known to be essential for MTB virulence and interaction with the host immune system^30^.

In addition to environmental and host-related factors, our study suggests that bacterial factors must also be considered when aiming to understand major transmission events. WGS analysis coupled with a simple in vitro infection model may provide a rapid screening platform of closely related variants with a differential transmission success. Once the involvement of bacterial factors is shown, additional studies on the candidate genes and polymorphisms should be required to more thoroughly characterize them and finally define determinants of MTB virulence, transmissibility, or evasion of the immune system. Targeting these SNPs with tailored allele-specific PCRs may allow fast tracking of strains requiring special surveillance.
